# NME proteins regulate class switch recombination

**DOI:** 10.1002/1873-3468.13290

**Published:** 2018-11-23

**Authors:** Simin Zheng, Anthony Kusnadi, Jee Eun Choi, Bao Q. Vuong, Daniela Rhodes, Jayanta Chaudhuri

**Affiliations:** ^1^ Immunology Program Memorial Sloan Kettering Cancer Center New York NY USA; ^2^ Immunology and Microbial Pathogenesis Program Weill Cornell Graduate School of Medical Sciences New York NY USA; ^3^ NTU Institute of Structural Biology Nanyang Technological University Singapore Singapore; ^4^ Arthritis and Tissue Degeneration Program and Genomics Center Hospital for Special Surgery New York NY USA; ^5^ Department of Biology City College of New York NY USA

**Keywords:** DNA recombination, G‐quadruplex, protein–DNA interaction

## Abstract

Class switch recombination (CSR) in B cells involves deletion‐recombination at switch (S) region DNA and is important for the diversification of antibody isotypes during an immune response. Here, we identify two NME [NM23/NDPK (nucleoside diphosphate kinase)] isoforms, NME1 and NME2, as novel players in this process. Knockdown of NME2 leads to decreased CSR, while knockdown of the highly homologous NME1 results in increased CSR. Interestingly, these NME proteins also display differential occupancy at S regions during CSR despite their homology; NME1 binds to S regions prior to stimulation, while NME2 binds to S regions only after stimulation. To the best of our knowledge, this represents the first report of a role for these proteins in the regulation of CSR.

## Abbreviations

**AID**, activation‐induced cytidine deaminase

**CSR**, class switch recombination

**DSBs**, DNA double‐strand breaks

**NME**, nucleoside diphosphate kinase

**SEC**, size exclusion chromatography

Immunoglobulin (Ig) heavy‐chain (Igh) class switch recombination (CSR) is an important process to diversify antibody function during an immune response. Upon encounter with antigens, mature B cells can undergo CSR, where deletion‐recombination occurs to replace the default Cμ constant region gene (C_H_) with one of the several downstream C_H_ segments (Cγ, Cε, or Cα). During this process, activation‐induced cytidine deaminase (AID) deaminates cytosines in the repetitive DNA elements called switch (S) regions that precede each C_H_ gene segment. Subsequent recruitment of the ubiquitous base‐excision and mismatch repair machineries to the deaminated DNA results in formation of DNA double‐strand breaks (DSBs) in the S regions. Repair of DSBs between the donor (Sμ) and a downstream acceptor S region deletes the intervening DNA and couples a new C_H_ gene to the variable region gene segment, thus allowing the B cell to ‘switch’ from expressing IgM to one producing either IgG, IgE, or IgA, with each isotype having a distinct function during an immune response 1.

While the generation of DSBs in S regions is essential for CSR, the molecular mechanisms involved are still not fully understood. Furthermore, not all the players involved in their generation and/or repair have been identified. Thus, in an effort to identify novel players that might play a role in the process, we examined the proteins that bind to DSBs in a B cell line, CH12, stimulated to undergo CSR. Using a reverse ChIP proteomic screen that involved *in situ* biotinylation of DSBs, followed by pull down of biotinylated DNA fragments and determination of associated proteins by mass spectrometry, we have identified NME [NM23/NDPK (nucleoside diphosphate kinase)] isoform 2 (NME2) as a protein that binds to DSBs.

NME2 is a member of a family of proteins well‐characterized for their housekeeping function in catalyzing phosphoryl transfer to nucleoside diphosphates during the synthesis of nucleoside triphosphates 2. However, it is becoming increasingly clear that these NME proteins have other roles in the cell as well and their activity on DNA is of particular interest for CSR. For instance, the highly homologous isoform, NME1, can bind to DNA and exhibit nuclease activity 3. NME2 has further been reported to bind to telomeric DNA 4 and G‐quadruplex (G4) DNA 5. The repetitive nature of telomeric DNA resembles that of S regions, and G4 structures formed by switch sequences have been implicated in CSR 6, 7. Thus, we investigate if NME proteins play any roles in CSR.

Knockdown of NME2 resulted in reduced CSR in the CH12 B cell line. Surprisingly, knockdown of the closely related isoform, NME1, in CH12 cells led to a different outcome and increased CSR instead. NME1 and NME2 also exhibited differential occupancy at S regions during CSR; NME1 binds to Sμ in unstimulated cells, while NME2 binds to Sμ only upon stimulation for CSR. Together, our results suggest that these NME proteins have different roles in CSR.

## Materials and methods

### Cell culture

CH12 cells were cultured and stimulated with anti‐CD40, IL‐4, and TGF‐β to induce CSR to IgA as described 8. Primary splenic B cells were purified from wild‐type BALB/c mice and stimulated with anti‐CD40 and IL‐4 to induce CSR to IgG1 (and IgE) as described 9.

### Antibodies

Antibodies for flow cytometry: anti‐IgA‐FITC (C10‐3). Antibodies for ChIP and immunoblot: anti‐γH2AX (JBW301) (Upstate, Lake Placid, NY, USA), anti‐NME1 (MC‐382) (Kamiya Biomedical Company, Tukwila, WA, USA), anti‐NME2 (MC‐412) (Kamiya Biomedical Company) or (1F2) (Abnova), anti‐ERH (1H4) (Abnova, Taipei, Taiwan), anti‐eIF5A (BD Transduction, San Jose, CA, USA), mouse IgG (I5381) (Sigma, St. Louis, MO, USA), anti‐AID 10.

### Reverse chromatin immunoprecipitation proteomic screen

The proteomic screen was adapted from 11. CH12 cells were stimulated for 48 h, following which live cells were harvest by Ficoll‐Hypaque separation and fixed with 1% formaldehyde at 37 °C for 10 min. Cells were resuspended in permeabilization buffer (100 mm TrisCl pH7.4, 50 mm EDTA, 1% Triton‐X‐100), incubated for 30 min on ice, then washed successively with cold PBS, water and 1× TdT buffer (Promega, Madison, WI, USA). 0.15 U·μL^−1^ TdT and 50 μm biotin‐11‐dUTP were added, and reaction was incubated at 37 °C for 30 min for the biotinylation of DSBs. EDTA (50 μm ) was added to stop the reaction and cells were washed with 100 mm Tris/HCl pH 7.4, 150 mm NaCl; followed by binding buffer (100 mm Tris/HCl, pH 7.4, 20% glycerol, 50 mm EDTA, 150 mm NaCl, 0.1% Triton‐X). Cells were resuspended in binding buffer at 20 × 10^6^ cells·mL^−1^ and sonicated on ice. Lysates were precleared by incubation with agarose beads and high speed centrifugation, after which streptavidin beads were added and incubated at 4 °C overnight. Streptavidin beads were washed twice with low salt buffer (20 mm Tris pH8, 150 mm NaCl, 2 mm EDTA, 1% Triton‐X, 0.1% SDS), twice with high salt buffer (20 mm Tris pH8, 500 mm NaCl, 2 mm EDTA, 1% Triton‐X, 0.1% SDS), LiCl buffer (10 mm Tris, 250 mm LiCl, 1 mm EDTA, 1% deoxycholic acid, 1% IGEPAL‐CA630), and twice with TE. To elute and reverse the formaldehyde crosslinks, beads were boiled for 45 min in 2× SDS loading buffer for protein analysis by mass spectrometry, or 2% SDS followed by phenol–chloroform extraction for DNA analysis by PCR.

### Chromatin immunoprecipitation

ChIP was performed as described 8. ChIP DNA was analyzed by PCR for Sμ and control loci (p53 and Iμ promoter) as described 12.

### Analysis of B cells

Mouse splenic B cells were harvested unstimulated (0 h), as well as 24, 48, 72, and 96 h poststimulation with anti‐CD40 and IL‐4. Expression of NME1 and NME2 proteins was determined by immunoblot. For measurement of NME1 and NME2 mRNA levels, cDNA was prepared by reverse transcription (RT) of 1 μg of RNA using the ProtoScript first strand cDNA synthesis kit (New England BioLabs, Ipswich, MA, USA), followed by qPCR using cDNA and PowerUp SYBR green master mix (Thermo Fisher, Waltham, MA, USA). qRT‐PCR values of NME1 and NME2 were normalized to β‐actin mRNA as the reference gene, and the 0 h reference sample. qPCR primers used include: NME1 (F: AGGACCAGTGGTTGCTATGG, R: TACAGAATCGCTGCCATGAA), NME2 (F: GCAGCATTACATCGACCTGA, R: GATGGTGCCTGGTTTTGAAT), β‐actin (F: TGCGTGACATCAAAGAGAAG, R: CGGATGTCAACGTCACACTT).

### Knockdown of NME proteins

Knockdown of NME1 and NME2 in CH12 cells was performed as described 8. Briefly, 293T cells were transfected with 12 μg lentiviral scrambled control shRNA (Addgene, Watertown, MA, USA), NME1 MISSION™ shRNA (TRCN000004732; Sigma) or NME2 MISSION™ shRNA vector (TRCN000005419; Sigma), 9 μg packaging vector psPAX2 (Addgene), and 3 μg envelope vector pMD2.G (Addgene) using Lipofectamine 2000 (Invitrogen, Carlsbad, CA, USA) according to the manufacturer's protocol. Transfection media was removed 16–18 h later and fresh media was added and collected as viral supernatant 24 h later. Polybrene was added to the viral supernatant at a final concentration of 8 μg·mL^−1^. About 4 mL viral supernatant was used to transduce 1 × 10^6^ CH12 cells by spinoculation at 2000 r.p.m., 25 °C for 2 h; after which, viral supernatant was replaced with CH12 growth media. Twenty‐four hours after transduction, infected CH12 cells were selected in 3 μg·mL^−1^ puromycin (Sigma) for 3 days, before stimulation with anti‐CD40, IL‐4, and TGF‐β for CSR (in puromycin containing media). Knockdown was determined by immunoblot using NME‐isoform‐specific antibodies.

### Germline transcripts and cell proliferation

Germline transcripts, Iμ‐Cμ and Iα‐Cα, were amplified using primers as described 13 and threefold serial dilutions of the cDNA template. β‐actin mRNA was used as the reference gene. Cell proliferation was assayed by staining cells with the cytoplasmic cell tracking dye, SNARF, followed by evaluation of the decrease in SNARF intensity over time in culture as described 8.

### Purification of NME2 protein

Recombinant NME2 protein was purified as described 5. Briefly, His_6_‐tagged NME2 protein was expressed in BL21DE3(RIPL) *Escherichia coli* induced with 1 mm IPTG at 37 °C for 4 h. Cells were lysed in lysis buffer (20 mm Tris, pH 8.0; 0.5 m NaCl, 20 mm imidazole, 1 mm TCEP, 0.5 mg·mL^−1^ benzonase) and the His_6_‐NME2 protein were purified using Ni‐NTA chromatography, followed by size exclusion chromatography (SEC) using HiLoad 16/60 Superdex 200 (GE Healthcare, Chicago, IL, USA) in SEC buffer (10 mm HEPES, pH7.5; 300 mm NaCl, 10% glycerol, 1 mm TCEP).

### Gel shift assay

The gel shift assay was adapted from 5. 5′‐FAM‐labeled single‐stranded DNA (ssDNA) oligonucleotides were purchased from Integrated DNA Technologies (Coralville, IA, USA). Oligonucleotides (10 μm) were folded in folding buffer (20 mm Tris pH 7.5, 100 mm KCl, 1 mm EDTA) by heating at 95 °C for 5 min, then allowed to cool passively to room temperature. Folded DNA (0.6 μm) was mixed with recombinant NME2 protein in reaction buffer (50 mm Tris pH 8.0, 100 mm KCl, 1 mm TCEP, 50 μg·mL^−1^ BSA) at room temperature for 30 min, followed by electrophoresis on 5% native polyacrylamide TBE gel in 0.5× TBE at 70 V for 1 h and analyzed using Typhoon FLA7000 scanner (GE Healthcare). For sequences of oligonucleotides used, see Fig. S3.

## Results

### NME2 binds to DSBs in S regions during CSR

**Figure 1:**
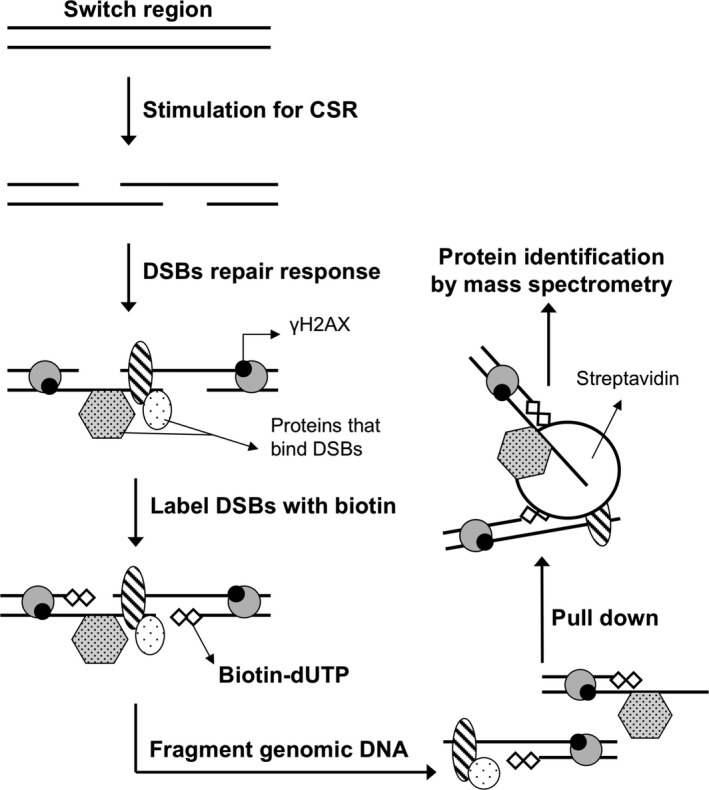
The reverse ChIP proteomic screen. Schematic representation of the proteomic screen to identify proteins associated with DSBs during CSR. Mouse B cell line CH12 was stimulated to undergo CSR. DSBs occur in switch regions and activates the DSBs repair response, including the phosphorylation of histone H2AX (γH2AX) and recruitment of proteins to the DSBs. The cells are fixed with formaldehyde and DSBs labeled with biotinylated nucleotides using terminal deoxynucleotidyl transferase (TdT). After labeling, TdT and excess nucleotides are removed and the DNA is fragmented by sonication. Biotinylated fragments are pulled down by streptavidin beads, followed by washes to remove nonspecific binding. Formaldehyde crosslinks are reversed by heat and DNA–protein complexes in the pull down are dissociated for identification of the proteins bound by mass spectrometry.

We have established a reverse ChIP proteomic screen adapted from 11 to identify proteins recruited to DSBs in S regions during CSR in CH12 cells (Fig. 1). The mouse CH12 B lymphoma cell line can be stimulated to undergo CSR in culture from IgM to IgA by a combination of anti‐CD40, interleukin 4 (IL‐4), and transforming growth factor β (TGF‐β) (henceforth referred to as CIT), and has been widely used as a model system to study CSR 7, 8, 14. Stimulated CH12 cells were fixed with formaldehyde and permeabilized to allow for *in situ* biotinylation of DSBs using terminal deoxynucleotidy transferase (TdT) (Fig. 1). Chromatin is then sheared, and biotinylated fragments with associated proteins are isolated on streptavidin beads and analyzed (Fig. 1).

**Figure 2:**
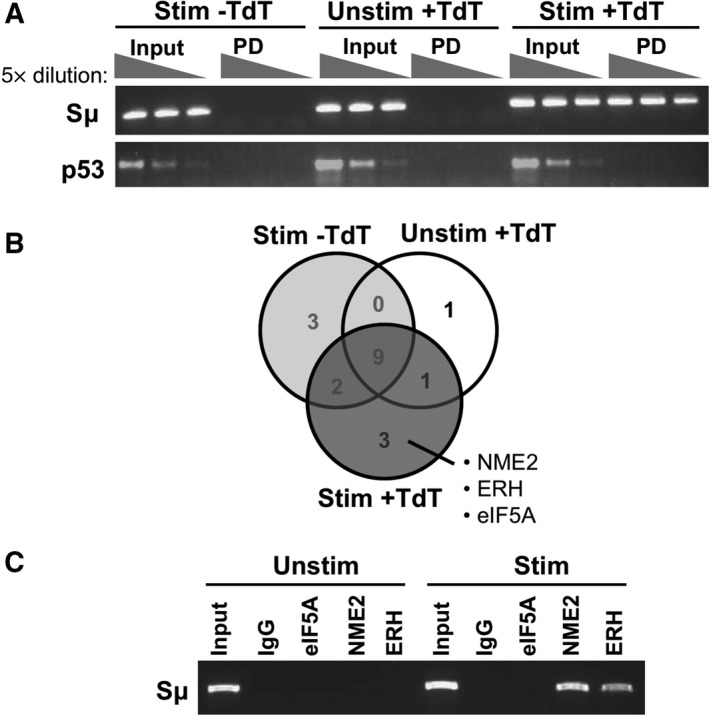
NME2 identified as a candidate protein that binds S regions upon stimulation. (A) Stimulation‐ and TdT‐dependent pull down (PD) of Sμ DNA fragments by the reverse ChIP proteomic screen is verified by PCR. (B) Three proteins (NME2, ERH, eIF5A) present only in stimulated and TdT‐labeled samples were identified as candidate proteins. (C) To validate candidate proteins, ChIP was performed on unstimulated CH12 cells or on CH12 cells 48 h after stimulation with CIT, using anti‐eIF5A, anti‐NME2, anti‐ERH, or control IgG antibodies. Sμ DNA in ChIP samples was detected by PCR.

As expected, the assay was able to recover Sμ DNA from stimulated CH12 cells labeled by TdT, but not without TdT‐mediated biotinylation, nor in unstimulated CH12 cells that contain few DSBs in S regions (Fig. 2A). DNA from the control locus p53 was not detected in any of the samples, indicating that the screen is specific for capturing DNA fragments near DSBs (Fig. 2A).

Proteomic analysis of the samples by mass spectrometry identified NME2, enhancer of rudimentary homolog (ERH) and eukaryotic translation initiation factor 5A (eIF5A) as proteins that are only present in stimulated and TdT‐biotinylated cells (Fig. 2B). NME2 and ERH were further validated to bind to S regions only upon stimulation for CSR, using ChIP assay and antibodies specific for each candidate protein (Fig. 2C). We chose to focus our studies on NME2, although the function of ERH is also of interest and warrant future explorations.

### NME2 binds to G‐quadruplex structures formed by S region DNA

The stimulation‐dependent binding patterns of NME2 to S regions led us to hypothesize that NME2 is recognizing features in the DNA locus that are altered by CSR. Upon stimulation for CSR, transcription occurs through S regions, resulting in the formation of RNA:DNA hybrid structures such as R‐loops 15. This exposes the guanine‐rich non‐template strand, which has the propensity to form G‐quadruplex (G4) structures 6. G4 structures are noncanonical motifs formed by guanine‐rich nucleic acids, whereby the guanine residues base pair though Hoogsteen interactions and stack upon one another to form a four‐stranded structure that is further stabilized by a central cation 16, 17. These G4 structures have been described to play important roles in many diverse areas of biology 18. As NME2 has been reported to bind G4‐DNA 5, 19, we examined if binding to these structures is a potential mechanism for the recruitment of NME2 to the S regions during CSR.

**Figure 3:**
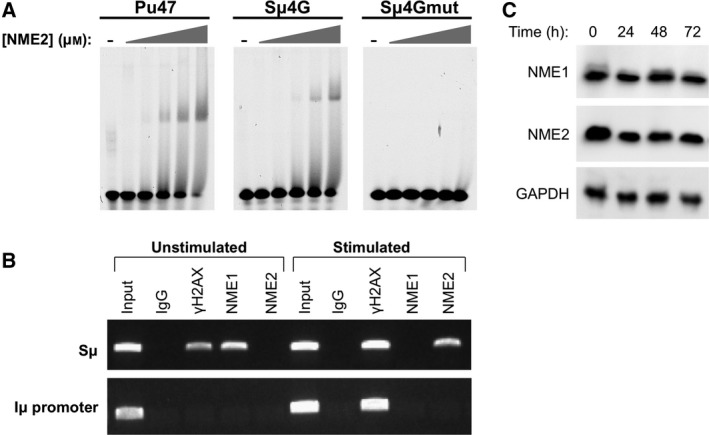
NME1 and NME2 exhibit differential occupancy at S region during CSR. (A) NME2 binds to G4‐forming S region DNA. Binding of NME2 to Pu47, Sμ4G, and mutant Sμ4G (Sμ4Gmut) ssDNA was determined by gel shift assay using 0.6 μm ssDNA and recombinant NME2 protein. Triangles indicate increasing concentration of NME2 protein (2.5, 5, 10, 20, 40 μm) used in the binding reaction. Data shown are representative of three independent experiments. (B) ChIP was performed on unstimulated CH12 cells or on CH12 cells 48 h after stimulation with CIT, using anti‐γH2AX, anti‐NME1, anti‐NME2, or control IgG antibodies. Sμ and control locus Iμ promoter DNA in ChIP samples were detected by PCR. (C) NME proteins expression was analyzed at indicated times following stimulation by immunoblot with anti‐NME1, anti‐NME2, and anti‐GAPDH (control) antibodies. Data shown are representative of three independent experiments.

To determine if NME2 binds to S region DNA in a G4‐dependedent manner, we performed gel shift assays using 6‐fluorescein amidite (FAM)‐labeled single‐stranded DNA (ssDNA) oligonucleotides and purified NME2. Recombinant His‐tagged NME2 protein was incubated with FAM‐labeled ssDNA and the reactions were analyzed by native polyacrylamide gel electrophoresis. As previously reported 19, NME2 bound to the well‐characterized G4‐forming Pu47 sequence that corresponds to the NHEIII_1_ region in the c‐myc promoter (Fig. 3A). Similarly, NME2 bound to Sμ4G, a ssDNA representing four Sμ repeats in tandem known to fold into G4 structures 7; while no binding was observed for its mutant form Sμ4Gmut, which does not form G4 structures 7 (Fig. 3A). Thus, NME2 can recognize G4 structures formed by S region DNA, suggesting that these structures may play a role to recruit NME2 to S regions during CSR.

### NME1 and NME2 exhibit differential occupancy at Sμ during CSR

NME2 shares 88% homology to NME1 at the protein level, and the two isoforms function as homo‐ and heterohexamers to catalyze the transfer of phosphates to nucleoside diphosphates 20. Despite being highly similar in sequence and their shared role in nucleoside metabolism, NME1 and NME2 have been reported to have different cellular functions 2, 20. This led us to examine the binding of NME1 to S regions during CSR. We performed ChIP using NME‐isoform specific antibodies and analyzed the samples for presence of Sμ by PCR. As observed previously during the validation of the proteomic screen (Fig. 2C), we detected NME2 binding at Sμ in stimulated CH12 cells but not in unstimulated cells (Fig. 3B).

Interestingly, NME1 exhibited a reverse binding pattern to NME2; NME1 localized to Sμ only in unstimulated CH12 cells but not when the cells were stimulated (Fig. 3B). Neither NME1 nor NME2 bound to the control locus Iμ promoter (Fig. 3B), demonstrating the specificity of these proteins for the S region. Increased presence of γH2AX at Sμ and the nearby Iμ promoter upon stimulation indicates increased DNA double‐strand breaks and serves as a marker for activation of CSR (Fig. 3B). Furthermore, stimulation did not affect the expression of NME1 and NME2 (Fig. 3C), suggesting that the changes in binding of these proteins to Sμ upon stimulation were not due to altered levels of protein. These results suggest that NME1 and NME2 localize differently to S regions in a stimulation‐dependent manner, suggesting that they may play distinct roles in CSR.

### NME proteins play complementary roles in CSR

**Figure 4:**
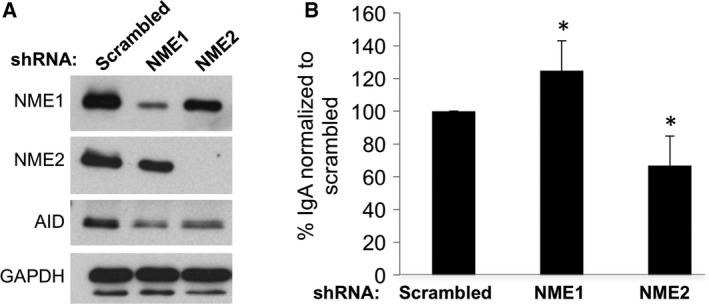
Knockdown of NME proteins affect CSR. CH12 cells were transduced with an shRNA against NME1, NME2, or scrambled control shRNA. Successful transductants were selected using puromycin, followed by stimulation with CIT for CSR. (A) Knockdown of NME proteins and expression of AID were determined by immunoblot using NME‐isoform specific, anti‐AID, and anti‐GAPDH (control) antibodies. Result shown is representative of three independent knockdown experiments. (B) CSR to IgA was assayed by flow cytometry 72 h after stimulation and normalized to the scrambled control. Data represent the mean of three independent knockdown experiments ± SD. *P < 0.05.

While NME1 and NME2 are expressed in mouse primary splenic B cells (Fig. S1), *ex vivo* splenic B cell cultures are short‐lived and experiments involving manipulations of these cells to study CSR have been technically challenging. Thus, to determine the function of NME proteins in CSR, we knocked down NME1 and NME2 independently in CH12 cells using shRNA, then stimulated the cells with CIT for CSR (Fig. 4A). The level of germline transcripts (Fig. S2A) and rate of proliferation (Fig. S2B) were not altered in the NME1 and NME2 knockdown cells compared to the scrambled shRNA controls cells. Thus, knockdown of NME1 and NME2 proteins did not have an overt effect on transcription and cell proliferation.

Upon stimulation, CSR of NME2 knockdown cells was reduced compared to the scrambled shRNA controls cells (Fig. 4B), indicating that NME2 plays a stimulatory role in CSR. Surprisingly, knockdown of the NME1 isoform (Fig. 4A) resulted in a significant increase in CSR instead (Fig. 4B), indicating an inhibitory role in CSR.

**Figure 5:**
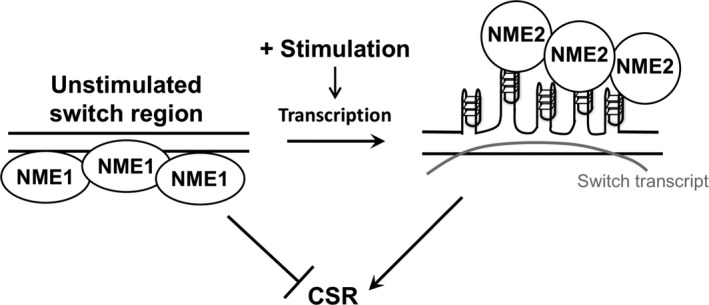
Model for the mechanism of NME proteins in the regulation of CSR. Prior to activation for CSR, NME1 binds to the S region and inhibits CSR. Upon stimulation, transcription occurs through the switch region, which allows the G‐rich single‐stranded S region DNA to form G4 structures. NME1 dissociates from the activated S region, while NME2 binds to the G4 formed in the S region to facilitate CSR. Hence, NME1 and NME2 act as a coordinated inhibitory–stimulatory pair to regulate CSR.

The stimulation‐dependent, opposing binding pattern of NME1 and NME2 to S regions (Fig. 3B) corroborates with their respective inhibitory and activating roles in CSR (Fig. 4). Taken together, these results suggest a model whereby differential localization of NME1 and NME2 to S regions function to regulate CSR. (Fig. 5).

## Discussion

Our results demonstrate that NME1 and NME2 proteins play opposing roles in CSR. The knockdown experiments indicate that NME1 inhibits CSR while NME2 promotes CSR (Fig. 4). This is surprising given the similarities between the two proteins and their well‐described common function in nucleoside triphosphate synthesis. However, in recent years, there is ever increasing evidence that the NME1 and NME2 isoforms have independent and unique roles in addition to their shared ones. For instance, NME1, but not NME2, mediates DNA degradation during granzyme A‐induced apoptosis 21. Conversely, NME2, but not NME1, has been reported to be able to activate G proteins 22. Thus, their different effects on CSR are yet another example of the distinct functions of the two isoforms.

While the exact mechanisms by which NME1 and NME2 mediate their opposing effects on CSR is still unclear, their stimulation‐dependent differential occupancy at S regions (Fig. 3) suggests several possibilities. NME1 is known to be a 3′–5′ exonuclease and function to maintain genome stability 3, 23. It is likely that DNA breaks could be resected by NME1 poised at the unstimulated S region to promote short‐range microhomology‐mediated repair that keep the switch region intact, instead of the long range nonhomologous end joining that would result in recombination. Thus, in this manner, NME1 might inhibit CSR.

Conversely, G4 structures formed in the non‐template strand of S regions upon stimulation might limit access of factors, such as RPA and AID 1, that are important for CSR. NME2 has been reported to have G4‐unwinding activity 5 and its recruitment to S regions upon stimulation might facilitate resolution of G4 structures, hence, allowing other factors to access the DNA in order to mediate CSR. It is becoming increasingly clear that G4 structures regulate many key biological functions 18, and it is likely that G4 and proteins that bind to them, such as NME2, are also important here in the process of CSR. Additionally, AID expression is slightly reduced in both NME1 and NME2 knockdown cells (Fig. 4A). While lower AID expression is unlikely to result in the increased CSR observed in NME1 knockdown cells, it might contribute to the CSR defect in NME2 knockdown cells. These and other possible mechanisms await further investigations.

It is interesting to note that unlike the effects observed with acute depletion of NME2 by shRNA knockdown (Fig. 4), chronic depletion in NME2‐knockout mice does not impair CSR 24. NME2^−/−^ mice have been reported to produce normal amounts of IgG1 antibodies in response to immunization with a T‐dependent antigen 24. The lack of CSR phenotype in the NME2^−/−^ mice indicates that one or more mechanisms might be activated during chronic loss of NME2 that preserves CSR. While these compensatory pathways have yet to be identified, it is beyond the scope of this study. Our study also highlights the need for both acute as well as chronic depletion studies to fully understand the role of genes in biological processes, and caution against the over‐reliance on a particular experimental model system. Several reports of the different outcomes of acute and chronic depletion further suggest that this is a prevalent theme in biology 25, 26. Chronic loss by deletion in knockout animals would allow us to characterize any redundant or compensatory pathways, while acute depletion studies would potentially unmask new players that are important to the process.

In conclusion, using a proteomic screen for factors that bind to DNA breaks, we have identified two novel factors that bind to the S regions. Despite their sequence similarities, NME1 and NME2 proteins act as an inhibitory–stimulatory pair that, through their stimulation‐dependent localization to S regions, regulates CSR (Fig. 5).

## Author contributions

SZ and JC conceptualized the study and designed the experiments. SZ performed most of the experiments and wrote the manuscript. AK provided technical support. JEC and BQV performed the time‐course expression studies in CH12 and mouse splenic B cells. JC and DR reviewed and edited the manuscript.

## Supporting information


**Fig. S1.** Mouse splenic B cells express NME1 and NME2.

**Fig. S2.** Knockdown of NME1 and NME2 do not affect germline transcription and proliferation.

**Fig. S3.** Sequences of single‐stranded DNA oligonucleotide used in the gel shift assay. Oligonucleotides were synthesized with a 5′‐FAM label and a triple thymine linker, followed by the sequence of study.
