# Supplementary figures and images for: NME proteins regulate class switch recombination
Source: FEBS Lett. 2018 Nov 23;593(1):80–7. doi: 10.1002/1873-3468.13290 (PMC6333498; doi:10.1002/1873-3468.13290)

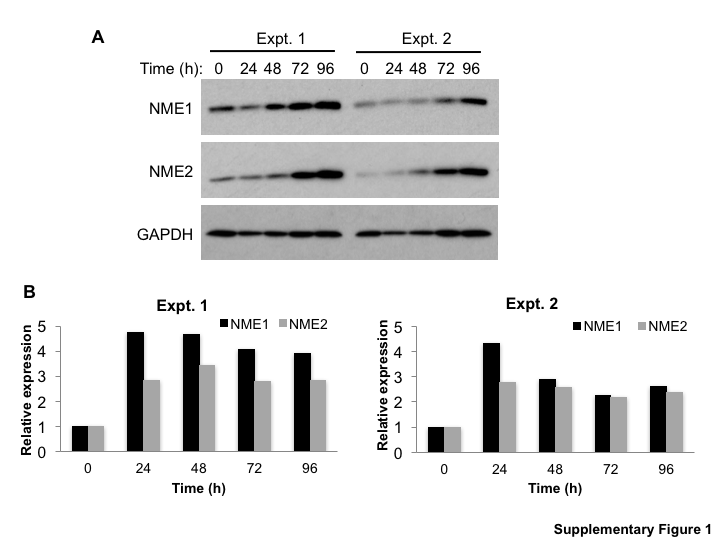

Supplement: Supplementary file 1 — Fig. S1. Mouse splenic B cells express NME1 and NME2. [file FEB2-593-80-s001.tiff]

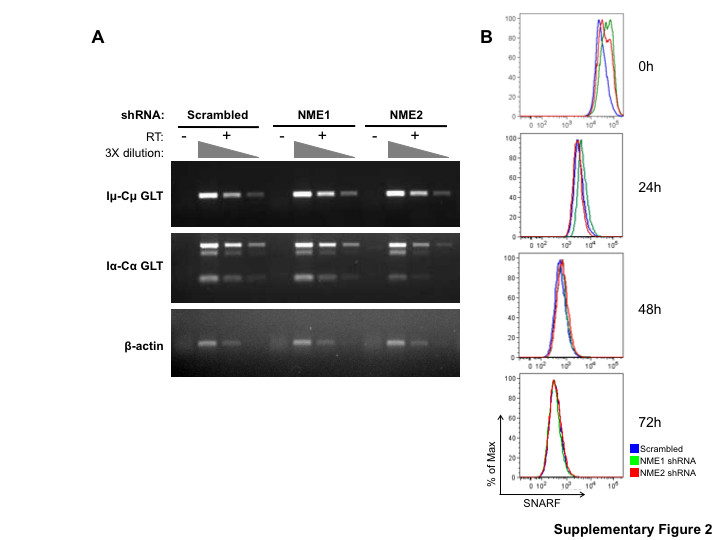

Supplement: Supplementary file 2 — Fig. S2. Knockdown of NME1 and NME2 do not affect germline transcription and proliferation. [file FEB2-593-80-s002.tiff]

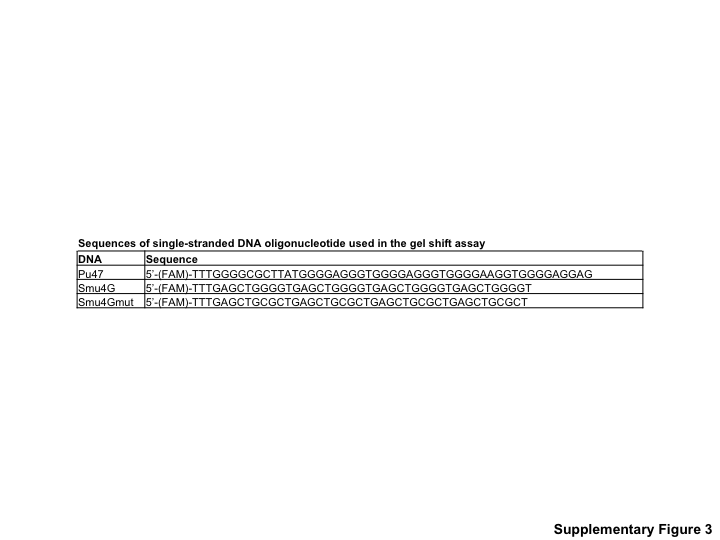

Supplement: Supplementary file 3 — Fig. S3. Sequences of single‐stranded DNA oligonucleotide used in the gel shift assay. Oligonucleotides were synthesized with a 5′‐FAM label and a triple thymine linker, followed by the sequence of study. [file FEB2-593-80-s003.tiff]
